# Evaluation of vitamin D receptor expression in uterine leiomyoma and nonneoplastic myometrial tissue: a cross‐sectional controlled study

**DOI:** 10.1186/s12958-021-00752-x

**Published:** 2021-05-05

**Authors:** Maria Simone Oliveira Lima, Benedito Borges da Silva, Menandro Lima de Medeiros, Alesse Ribeiro dos Santos, Emerson Davi do Nascimento Brazil, Walberto Monteiro Neiva Eulálio Filho, Jerusia Oliveira Ibiapina, Antônio Gilberto Albuquerque Brito, Pedro Vitor Lopes Costa

**Affiliations:** Department of Gynecology, Federal University of Piauí, Rua Doutor Anisio Maia, 1261, Ininga, Piauí 64049-810 Teresina, Brazil

**Keywords:** Vitamin D receptor, Myoma, Nonneoplastic myometrium, Immunohistochemistry

## Abstract

**Background:**

Uterine myomas are the most frequent benign solid pelvic tumors in women of reproductive age. At present, uterine myomas are the most common indication for hysterectomy because of the morbidity they cause, including intense bleeding, compression of adjacent organs, pain, and infertility. Some studies show that vitamin D receptor (VDR) expression is correlated with the etiology of uterine leiomyomas. This study aimed to assess the expression of VDR in uterine leiomyoma and nonneoplastic myometrial tissue.

**Methods:**

A controlled cross-sectional study involving 40 women who underwent abdominal hysterectomy the Department of Gynecology of the Getúlio Vargas Hospital of Federal University of Piauí, Brazil, was performed to compare the immunohistochemical expression of VDR in samples of uterine leiomyoma tissue with adjacent nonneoplastic myometrial tissue. The mean percetages of stained nuclei in the two groups was compared by Student’s t teste, with significance established at *p* < 0.05.

**Results:**

The percentage of cells with nuclei stained by anti-VDR in the myometrial and leiomyoma tissue was 79.52 % (± 4.32) and 60.22 % (± 7.24), respectively (*p* < 0.0001).

**Conclusions:**

The mean percentage of nuclei expressing VDR was significantly lower in the uterine leiomyoma than in nonneoplastic myometrial tissue.

## Introduction

Uterine leiomyomas are the most frequent benign solid pelvic tumors in women of reproductive age [1]. They are usually detected between 30 and 40 years of age [2]. Furthermore, they may be related to biological changes in growth and development and are generally influenced by sex hormones, primarily estrogen [3]. Leiomyomas can have a significant impact on women’s quality of life. Depending on their anatomical position, quantity, and size, they can cause symptoms such as heavy bleeding, pain, compression of adjacent organs, and infertility [4]. They may also cause adverse gestational outcomes, such as miscarriages, fetal anomalies, and an increase in the indication for cesarean Secs. [5, 6].

Uterine leiomyomas are characterized by excessive extracellular matrix (ECM) deposition as well as an increase in cell proliferation [7]. Changes in this process of degradation and remodeling may contribute to the genesis of leiomyomas. The etiology of leiomyomas has not yet been fully clarified, and recently, it has been suggested that vitamin D can play an important role in their development [8]. The active metabolite of vitamin D, recognized as a steroid hormone, has been shown to be an inhibitor of cell proliferation and extracellular matrix production in fibroid tissue, thus reducing the volume of leiomyomas [9, 10].

Vitamin D exerts its effects by activating its cell receptor, vitamin D receptor (VDR), which consequently changes the transcription rates of the target genes responsible for various biological responses [10]. Such effects include immunomodulatory, antiproliferative, differentiating, and antineoplastic activities that may lead to actions such as the inhibition of cell proliferation, the induction of differentiation, and the induction of apoptosis. Furthermore, it can lead to protection from the malignant transformation of cells as well as the inhibition of cell growth [11–13].

Recent studies have evaluated the expression of VDR in both the myometrium and endometrium of the human uterus. Feng et al. [14] conducted a study to determine the expression of VDR in uterine leiomyoma tissue. Samples of myoma and myometrial tissue from different sites in five women who underwent hysterectomy were evaluated, and high VDR expression was observed in the central part of the leiomyoma samples compared to the myometrial samples. The expression of VDR in the leiomyoma margin did not differ from that in the central region of the lesion or the adjacent myometrium, but different levels of VDR expression were found depending on the location of the lesion, and a biological role of vitamin D signaling in the pathology leiomyoma was suggested. Another more recent study conducted by Al-Hendy et al. [15] evaluated the role of 1.25 (OH) 2D3 in the expression of sex steroid receptors in uterine fibroid cells in 14 patients, where they identified that the expression of VDR was lower in the fibroid tissue in 60 % of the cases (8) than in the adjacent myometrium.

Evidence shows that the expression of VDR in most cells could be an important cellular biomarker associated with the etiology of uterine leiomyomas [16]. However, there have been few studies evaluating the expression of this biomarker in uterine leiomyomatosis. Thus, the present study aimed to evaluate VDR expression in uterine leiomyomas and in adjacent nonneoplastic myometrial tissue.

## Patients and methods

This was a controlled cross-sectional study involving 40 Brazilian women who attended the Gynecological Department of the Getúlio Vargas Hospital of Federal University of Piauí, Brazil, from April to December 2017. The woman included in this study were of reproductive age (30 to 45 years; mean age 40.7 years), had symptomatic uterine leiomyomatosis and had undergone abdominal hysterectomy surgery. The exclusion criteria for the study were a postmenopausal status, hepatic, metabolic, cardiovascular, and renal diseases, reports of other types of malignancy, and previous hormonal or surgical treatment for leiomyoma. In addition, the following data were collected from medical records: age, parity, ethnicity, body mass index (BMI), fibroid size and uterine volume.

Regarding parity, 35 patients had had children and 5 had had no pregnancies. Regarding ethnicity, 27 patients were Afro-Brazilian and 13 were white. The mean body mass index was 26.26 (21.3–32.9). In particular, 17 (42.5 %) patients had a normal weight (19–24.9), 17 (42.5 %) were overweight (25–29.9), and 6 (15 %) were obese. The mean myoma size was 7.61 cm, ranging from 1.3 to 83.1 cm. The mean uterine volume was 370.2 cm^3^, ranging from 76.8 to 1438.0 cm^3^ (Table 1).

**Table 1:** Patient’s characteristics

	N	%
**Age**		
30–39	14	35
40–45	26	65
**Parity**		
Yes	35	87.5
No	5	12.5
**Ethinicity**		
White	13	33
Afro-Brazilian	27	67
**Body Mass Index**		
Eutrophic	17	42.5
Pre-obese	17	42.5
Obese	6	15
**Myoma size (cm)**		
Less than 5	22	55
5–10	15	37
More than 10	3	8
**Uterine Volume (cm**^**3**^**)**		
Less than 160	6	15
161–500	28	70
More than 500	6	15

Tissue samples of uterine leiomyomas and nonneoplastic myometrial tissue of approximately 1 cm in diameter were collected. Nonneoplastic tissue samples were collected from uterine regions clear of fibroids and at least 5 cm away from any myoma at the time of the abdominal hysterectomy procedure. Tissue samples were fixed in a 10 % buffered formalin solution for a period of 12–24 h. Then, all histological procedure steps were performed and the samples were embedded in paraffin. These samples were cut into 3 μm-thick sections. Some of the sections were stained with hematoxylin and eosin to confirm the diagnosis of uterine leiomyomatosis and identification of normal myometrial tissue, while other samples were reserved for immunohistochemical analysis.

In the immunohistochemical analysis, a rabbit anti-vitamin D3 receptor polyclonal antibody (orb214726, Biorbyt, UK), was used to stain for VDR at a dilution of 1:25 with bovine serum albumin (BSA). These sections were deparaffinized in xylol for 5 min, dehydrated in absolute ethanol and washed in buffered saline solution at pH 7.4 for 5 min. Then, the sections were treated for 5 min with 3 % hydrogen peroxide (H2O2) diluted in buffered solution to block the endogenous peroxide. For antigen recover, the slides were placed in racks containing 0.21 % citric acid (pH 6.0) and heated in a microwave oven for 15 min at maximum power. Phosphate-buffered saline containing Tween (PBS-Tween) was added to the slides after they had been cooled for 20 min. The tissue samples were incubated with specific primary antibody overnight at 4ºC, then the slides were subjected to three washes in PBS, dried and incubated with “In Vision TM System (DAKO, Code K 1672)” for 1 h at 37º C. Once again, the slides were washed in running water, distilled water and stained with Mayer’s hematoxylins for 30 s. The cuts were embedded with 100 % alcohol and xylol for dehydration and then placed in coverslips and Entellan resin. Finally, the slides were examined by light microscopy. Formalin-fixed and paraffin-embedded human lung cancer tissue stained with VDR antibody (dilution at 1:200) was used for positive control.

Quantitative biomarker expression was evaluated by two observers who were blinded to the sample types. These observers quantitatively counted the cells with positively stained nuclei (under 400× magnification) using an optical microscope connected to a video camera. For the quantification of VDR expression, a minimum of 500 cells were counted in each slide, whether stained with the anti – VDR antibody or not, at a magnification of 400 X beginning with the area in which VDR expression was greatest. In each case, the percentage of stained cells was obtained as the ratio of the number of cells with stained nuclei to number of cells multiplied by 100.

The results in the two groups were analyzed as the mean and standard error using Student’s t-test, and the average percentages of cells stained in the myoma and myometrial tissue were analyzed descriptively using boxplots. To verify the normality of the data, the Shapiro-Wilk test was performed. The correlation of the percentage of cells stained in the fibroid with the tumor size and uterine volume was examined with the Spearman coefficient. *P* < 0.05 was considered statistically significant. The Internal Review Board of the Federal University of Piauí approved the study, and all patients signed the free informed consent form prior to the study.

## Results

**Fig. 1:**
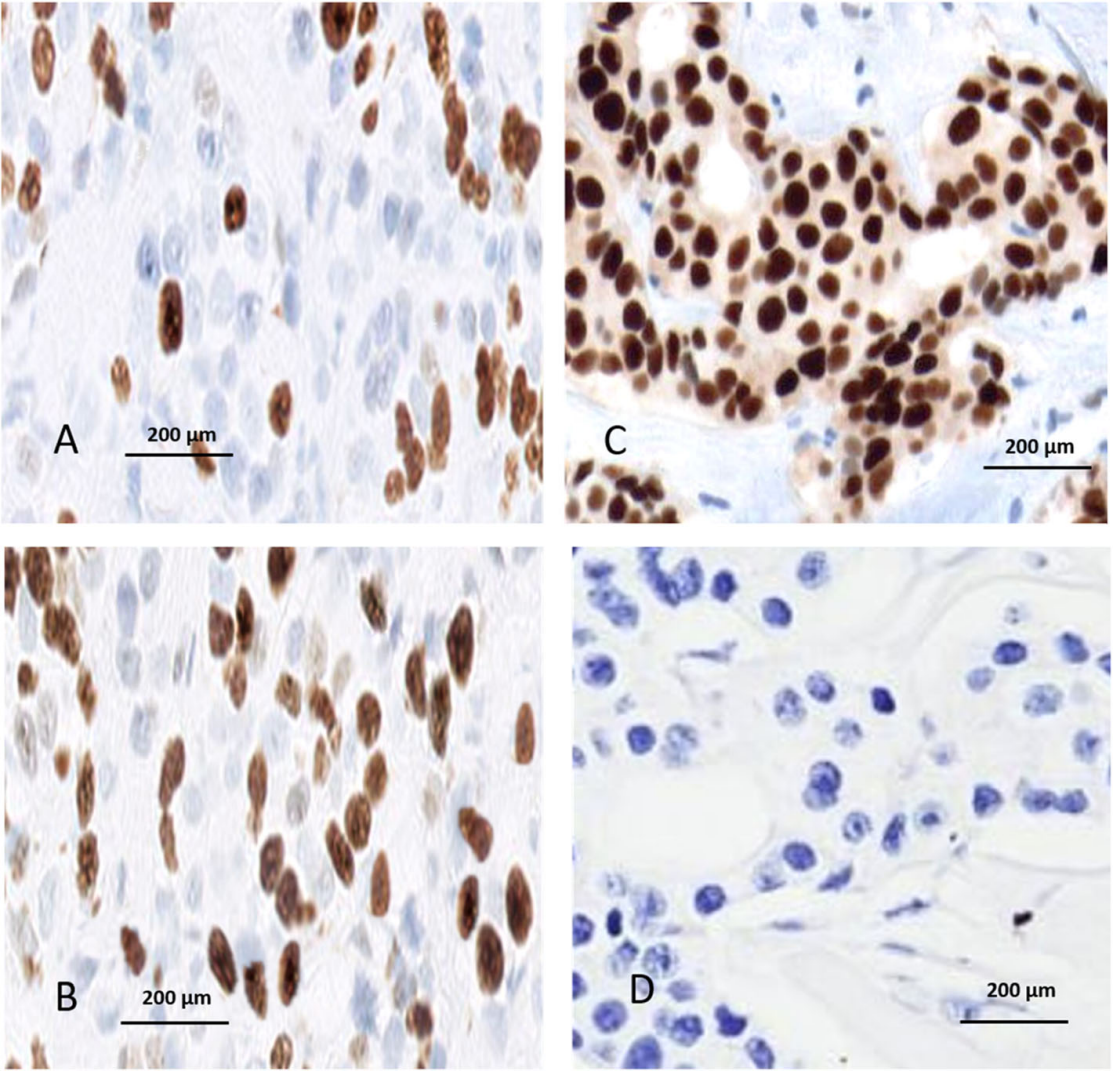
Photomicrograph of a histological section of a uterine leiomyoma showing sparse brown nuclear-staining by the anti-VDR antibody (**a**) compared to the extensive brown nuclear-staining with strong reativity to VDR in the adjacent normal myometrium (**b**). Positive control (**c**) and negative control (**d**). The counterstaining used was hematoxylin

**Fig. 2:**
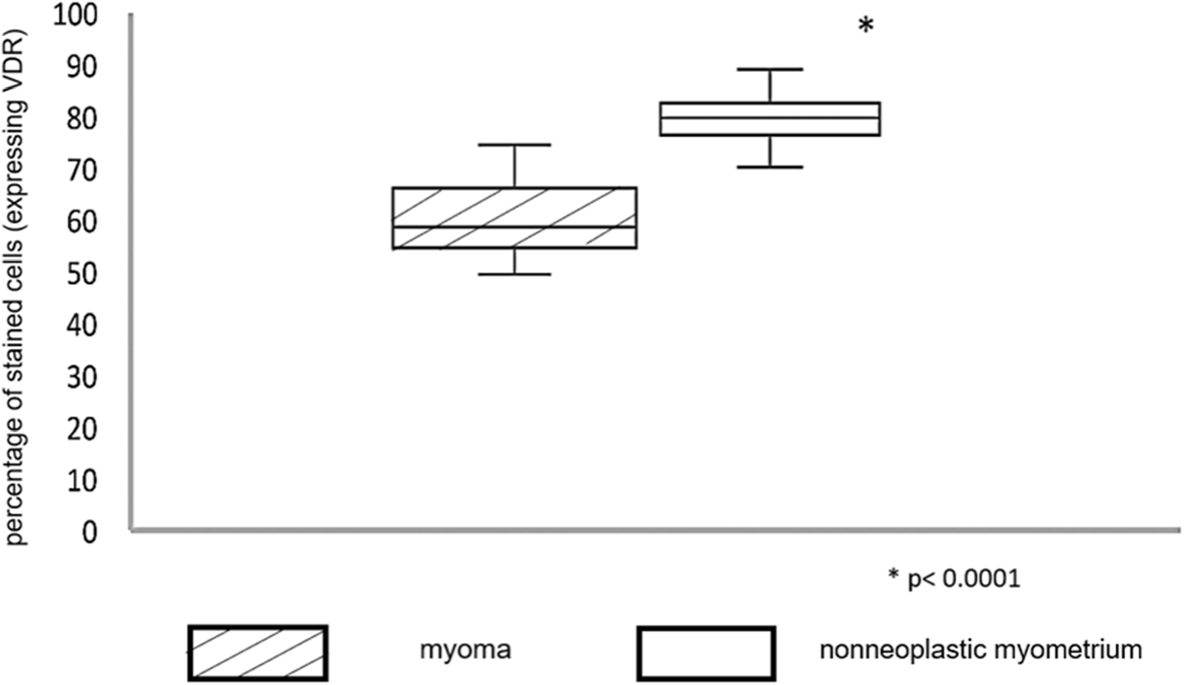
Box plot of the percentage of stained cells (expressing VDR) in myoma and nonneoplastic myometrial tissue

 Under light microscopy, we observed a scarcity of brown nuclei stained by anti-VDR antibody in the fibroma samples and many nuclei stained in the adjacent normal myometrial samples (Fig. 1). The mean percentage and standard deviation of VDR expression was 79.52 ± 4.32 in the myometrial tissue and 60.22 ± 7.24 (*p* < 0.0001) in the leiomyoma tissue, as described in Table 2 and represented in Fig. 2. There was no statistical correlation of expression of vitamin D receptor expression in uterine leiomyoma tissue with age, parity, tumor size, uterine volume or nutritional status (Table 3).

**Table 2:** Mean percentage of nuclei stained by anti-vitamin D receptor antibody in myoma and in nonneoplastic myometrial tissue

Group	N	Mean	Standard error	Median	Minimum	Maximum	***p***-value*
Control							
(1)	40	79.52	4.32	79.91	70.00	89.00	0.0001
Study							
(2)	40	60.22	7.24	58.55	50.00	75.00	

Source: The Author

1-Myometrium; 2-Myoma

*p-value of the t-test for equality of means

**Table 3:** Correlation between vitamin D receptor expression in uterine leiomyomas and clinical variables

Variables	correlation coeficiente (r)^a^	*p*
Age	0.116	0.477
Parity	0.093	0.567
Tumor size	0.129	0.428
Uterine volume	-0.067	0.682
Nutricional status	-0. 078	0.634

^a^correlation coeficiente of Spearman

## Discussion

In this study, we evaluated the expression of the biomarker VDR in human uterine leiomyoma tissue and compared it that in nonneoplastic myometrial tissue. Studies demonstrating the identification of VDR expression in most cells and the ability of some cells to produce active forms of vitamin D have suggesting an influence of this vitamin on the pathogenesis of uterine fibroids [8, 9, 17, 18].

The results of the present study demonstrate reduced VDR expression in leiomyoma tissue compared with myometrial tissue,suggesting an association with the pathogenesis and development of human uterine leiomyomatosis. The cause of the reduction in the expression of these receptors is still unknown, and it is not clear whether it occurs as an event related to the initiation or progression of uterine leiomyomas. A study by Halder et al. [8] corroborates our results. They evaluated the risk of benign uterine tumors associated with reduced levels of VDR protein in 40 patients and determined the biological function of 1.25 (OH) 2D3 in the regulation of proteins associated with the extracellular matrix, which are fundamental in the formation of leiomyomas, and found reduced VDR levels in more than 60 % of the uterine tumors analyzed compared with the adjacent myometrium. In their study, the levels of VDR in the uterine myoma samples were significantly lower than the levels in the adjacent myometrial samples, similar to the results of the present study.

Increased proliferation and Wnt/β-catenin pathway deregulation play a role in the development and growth of leiomyomas. Vitamin D exerts an antiproliferative effect on HULP cells through cell growth arrest and Wnt/β-catenin pathway inhibition [19]. The reduction in VDR expression would be a factor reducing the antiproliferative activity of vitamin D, thereby contributing to the pathogenesis of uterine leiomyomas.

A large cross-sectional study was conducted involving 21,746 women between 15 and 49 years of age from seven countries, Brazil, Canada, France, Germany, Italy, South Korea, and the United Kingdom, and women who were between 18 and 49 years of age from the United States. The authors found a prevalence of myomas of 7 % among Brazilian women [20]. According to the literature, the incidence of myomas is 2–3 times higher in black populations and are more often symptomatic, larger, and multiple in nature [21]. In the present study, all the evaluated women were of reproductive age; therefore, they had high hormone levels. Additionally, 67 % were Afro-descendants, thus presenting favorable characteristics for the etiology of the these uterine tumors.

In the etiology of myomas, in addition to the genetic, growth, and hormonal factors involved, other factors include age, family history, obesity, arterial hypertension, nulliparity, and diet (red meats, alcohol, caffeine, etc.) [22]. Obesity is associated with the increased secretion of adipokines, which significantly influence the growth and proliferation of tumor stroma and malignant cells [23]. In our study, 57.5 % of the women were overweight, and five of them were nulliparous.

Al-Hendy et al. [15], who researched the role of 1.25 (OH) 2D3 in the expression of sex steroid receptors in leiomyoma cells, realized that the deregulation of steroid hormones and their receptors could be a primary factor of myoma growth since 1.25 (OH) 2D3 VDR expression but acts as an antiestrogenic agent in these cells. Moreover, they showed a significant decrease in estrogenic receptor levels in leiomyoma cells treated with 1.25 (OH) 2D3 and analyzed for receptor expression and location (*p *< 0.05). In contrast, 1.25 (OH) 2D3 induced the expression of its own VDR, suggesting that 1.25 (OH) 2D3 acts as an antagonist of hormone receptors with antiestrogenic and antiprogesteronic functions.

Furthermore, Halder et al. [9] studied this antiestrogenic influence of vitamin D when they examined the effect of 1,25-dihydroxyvitamin D3 on fibrosis-related protein expression in TGF-3 induced uterine leiomyoma cells in vitro. Myoma cells were treated with TGF-3 with or without vitamin D. They identified that TGF-3 induced the expression of fibronectin and collagen protein type 1 in myoma cells, and this effect was suppressed by vitamin D, which was considered an antifibrotic factor in the treatment of benign uterine myomas.

Paffoni et al. [18] studied the serum levels of vitamin D in women with myoma and revealed that the vitamin D concentration was significantly lower in women with myomas than in women in the control group (11.1 and 18.0 ng/ml, respectively; *p* < 0.010 and OR = 2.2). Similar results were obtained by Baird et al. (2013), who evaluated vitamin D and the risk of uterine myomas and found that women with sufficient vitamin D had an estimated 32 % reduction in the incidence of myomas compared to those with insufficient vitamin D.

In addition, Sabry et al. [24] investigated whether low serum levels of vitamin D are correlated with increases in the risk and occurrence of uterine myomas and found that reduced serum levels of 25-(OH) vitamin D were significantly associated with the occurrence of myomas. A statistically significant inverse correlation was also observed between the serum levels of 25-(OH) vitamin D and the total leiomyoma volume within the case cohort.

The above studies indicate that the loss of vitamin D functions due to the reduction of vitamin D3 levels and/or reduced expression of VDR may be associated with the growth and development of several types of neoplastic lesions. Our results reinforce the hypothesis that low VDR expression may be associated with the growth and development of myomas, presenting itself as an important biomarker in this pathology. However, further studies are needed to assess the real importance of VDR expression in the etiopathogenesis of uterine leiomyomas.

## Conclusions

In conclusion, we observed significantly reduced VDR expression in human uterine leiomyomas compared with in nonneoplastic myometrial samples.

## Data Availability

The data and materials are stored in the HospitalGetulio Vargas of the Federal University of Piaui, Brazil.

## Abbreviations

BSA: Bovine Serum Albumin
ECM: Extracellular Matrix
VDR: Vitamin D Receptor
